# MDA-7/IL-24 suppresses human ovarian carcinoma growth in vitro and in vivo

**DOI:** 10.1186/1476-4598-6-11

**Published:** 2007-02-02

**Authors:** Began Gopalan, Manish Shanker, Sunil Chada, Rajagopal Ramesh

**Affiliations:** 1 Department of Thoracic and Cardiovascular Surgery, Unit 445, The University of Texas M. D. Anderson Cancer Center, 1515 Holcombe Boulevard, Houston, TX 77030, USA; 2 Introgen Therapeutics, Inc., 2250 Holcombe Boulevard, Houston, TX 77030, USA

## Abstract

**Background:**

Previous studies showed that the human melanoma differentiation-associated gene-7 (mda-7), also known as interleukin-24 (IL-24), has potent antitumor activity against human and murine cancer cells. However, the majority of these studies were limited to in vitro testing. In the present study, we investigated the antitumor activity of mda-7/IL-24 against human ovarian cancer cells both in vitro and in vivo.

**Results:**

In vitro, treatment of ovarian cancer cells with an adenoviral vector carrying the mda-7 gene (Ad-mda7) resulted in inhibition of cell proliferation and induction of cell cycle arrest, leading to apoptosis. We did not observe inhibitory activity in Ad-mda7-treated normal cells. In vivo, treatment of subcutaneous tumor xenografts with Ad-mda7 resulted in significant tumor growth inhibition when compared with that in control groups (*p *< 0.001). Molecular analysis of ovarian tumor tissue lysates treated with Ad-mda7 showed that MDA-7 protein expression was associated with activation of the caspase cascade.

**Conclusion:**

Our results show that treatment of ovarian cancer cells with mda-7/IL-24 results in growth suppression both in vitro and in vivo.

## Background

Ovarian cancer is the second most common gynecological malignancy in the United States and is the fifth leading cause of death in women worldwide [1]. An estimated 25,000 women have been diagnosed with ovarian cancer in 2005, and 50% of these patients have died of the disease. Despite advances in the treatment of ovarian cancer, the overall long-term survival rate of this disease has not improved significantly. Hence, developing and testing new therapeutic agents and strategies for it are warranted.

The human melanoma differentiation-associated gene-7 (mda-7), also known as interleukin-24 (IL-24), encodes a protein of 206 amino acids with a predicted molecular mass of 23.8 kDa [2]. Gene transfer studies have demonstrated that mda-7 exerts its antitumor activity in a spectrum of cancer cells via multiple cell-type-dependent signaling pathways, resulting in apoptosis [2,3]. Recent *in vitro *studies from our laboratory and those performed by others showed that mda-7/IL-24 can suppress ovarian cancer cell growth *in vitro *and is dependent on the Fas/FasL or stress activated p38 mitogen-activated protein kinase (MAPK) pathway [4,5]. Additionally, investigators have shown that an adenoviral vector carrying the mda-7 gene (Ad-mda7) radiosensitizes ovarian cancer cells *in vitro *[6]. Although mda-7/IL-24 has displayed antitumor activity against ovarian cancer cells *in vitro*, the documented evidence of this activity *in vivo *is limited.

In the present study, we investigated the antitumor properties of Ad-mda7 against human ovarian cancer cells both *in vitro *and *in vivo *in a mouse model. We demonstrated that Ad-mda7 selectively exerts its antitumor effects against ovarian cancer cells, leading to suppression of tumor growth *in vivo*.

## Results

### Ad-mda7 inhibits ovarian cancer cell proliferation, induces cell cycle arrest, and regulates signaling molecules associated with apoptosis

Prior to the start of our study, we determined the transduction efficiency of an adenoviral vector carrying green fluorescent protein in MDAH2774, OVCA420, and IOSE-80 cells at different doses. We observed a dose-dependent increase in transduction of both tumor and normal cells, as more than 60% of the cells were transduced at 3000 vp/cell (data not shown). Based on this result, we used Ad-luc and Ad-mda7 at 3000 vp/cell in all subsequent experiments.

**Figure 1:**
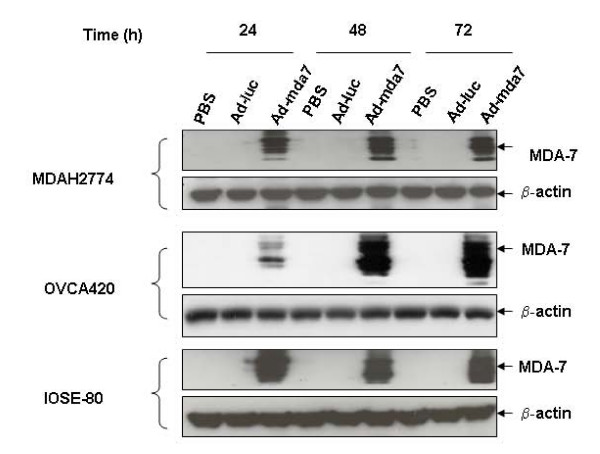
MDA-7 protein detection in Ad-mda7-treated cells. MDAH2774, OVCA420, and IOSE-80 cells treated with PBS, Ad-Luc, or Ad-mda7 were harvested at days 1, 2, and 3 after treatment and analyzed for exogenous MDA-7 protein expression by western blotting. MDA-7 expression was detected in Ad-mda7-treated cells but not in PBS-treated or Ad-luc-treated cells. β-actin was used as an internal loading control.

**Figure 2:**
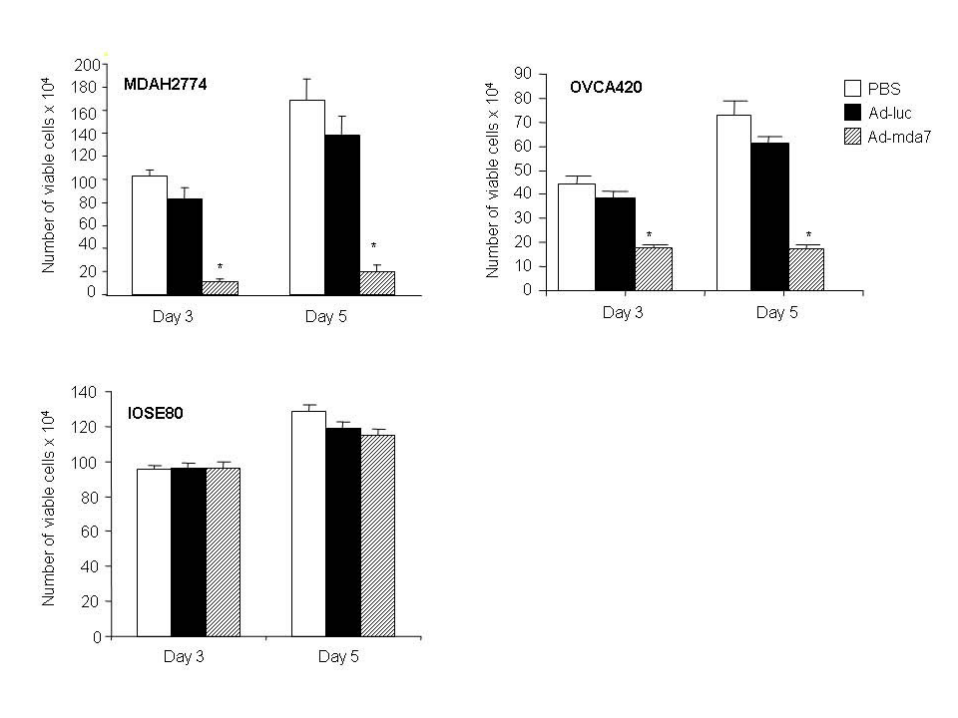
Ad-mda7 inhibits tumor cell proliferation. MDAH2774, OVCA420, and IOSE-80 cells treated with PBS, Ad-Luc, or Ad-mda7 were harvested at days 3, and 5 after treatment and analyzed for cell proliferation. Proliferation of tumor cells but not normal cells treated with Ad-mda7 was significantly lower (*p *= 0.001) than that of cells treated with PBS or Ad-Luc. Error bars denote standard error.

Treatment of MDAH2774, OVCA420, and IOSE-80 cells with Ad-mda7 resulted in exogenous MDA-7 protein expression that was detectable as multiple species on days 1, 2, and 3 after treatment (Figure 1). Detection of multiple species of MDA-7 protein in Ad-mda7-treated cells reflects glycosylated forms of the protein and in different stages of glycosylation. MDA-7 being a glycosylated protein has previously been reported [2,4,8]. MDA-7 protein expression was not detectable in cells treated with PBS or Ad-luc. To determine the effects of treatment with Ad-mda7 on cell proliferation, we treated cells with Ad-mda7 and examined them for the number of viable cells on days 3 and 5 after treatment. We observed significant inhibition of cell proliferation in Ad-mda7-treated ovarian cancer cells but not in PBS-treated or Ad-luc-treated cancer cells (*p *= 0.001; Figure 2). Also, we did not observe a significant inhibitory effect on cell proliferation in Ad-mda7-treated normal cells when compared with PBS-treated or Ad-luc-treated cells. These results showed that treatment with Ad-mda7 results in exogenous MDA-7 protein expression in both ovarian cancer and normal ovarian epithelial cells. However, MDA-7 selectively inhibits tumor-cell proliferation and minimally inhibits normal-cell proliferation. Our results concur with findings previously reported by us and others [2,3,7].

Previous studies from our laboratory and others showed mda-7/IL-24 inhibit human tumor cell proliferation by inducing cell cycle arrest at G2/M phase [2-8]. Based on these reports, we analyzed the effects of Ad-mda7 on the cell cycle at 72 h after treatment. Treatment of both ovarian cancer and normal cells with Ad-mda7 resulted in an increase in the number of cells at G2/M phase (Table 1). However, the number of cells at this phase was markedly higher in the cancer cells (50.2% and 29% of MDAH2774 cells and OVCA420 cells, respectively) than in normal cells (23%). Also, the number of Ad-luc-treated cancer cells was higher than that of PBS-treated cancer cells at G2/M phase. However, we did not observe a significantly higher number of Ad-luc-treated normal cells than PBS-treated normal cells at G2/M phase.

**Table 1:** Cell cycle distribution in ovarian cancer and normal cells treated with PBS, Ad-luc or Ad-mda7

	MDAH 2774 (%)			OVCA 420 (%)			IOSE 80 (%)		
	G1	*S*	G2M	G1	*S*	G2M	G1	*S*	G2M

PBS	72.2 ± 10.5	20.4 ± 9.3	7.3 ± 1.5	68.2 ± 4.9	21.7 ± 5.2	10.0 ± 1.6	40.2 ± 1.4	42.0 ± 2.6	18.0 ± 1.6
Ad-Luc	52.5 ± 6.2	25.2 ± 3.0	22.2 ± 3.3	68.2 ± 6.3	13.1 ± 2.3	18.2 ± 4.6	55.3 ± 2.2	26.3 ± 1.2	19.0 ± 1.5
Ad-mda7	243 ± 38	27.0 ± 14.6	50.2 ± 16.4	57.0 ± 4.8	14.4 ± 2.4	29.0 ± 4.2	48.8 ± 3.6	28.6 ± 2.1	23.0 ± 2.5

NOTE: Cells were treated with PBS, Ad-luc or Ad-mda7 for 72 hours followed by FACS analysis. The percentage of cells In each cell cycle phase was determined by analysis of the DMA content. Values are the means of duplicate experiments ± SD.

**Figure 3:**
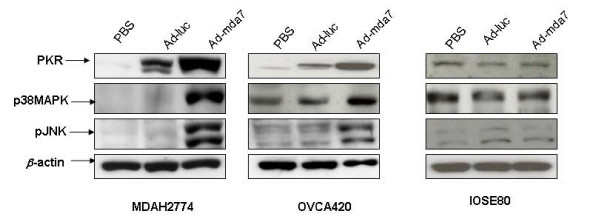
Ad-mda7 activates molecular markers associated with cell killing. Western blot analysis showed activation of PKR, p38MAPK, and pJNK in Ad-mda7-treated tumor cells but not in PBS-treated or Ad-luc-treated tumor cells. We did not observe activation of these markers in normal cells treated with PBS, Ad-luc, or Ad-mda7. β-actin was used as an internal loading control.

Previous studies also showed that cell signaling markers such as PKR, p38MAPK, and pJNK are activated in Ad-mda7-treated lung cancer and melanoma cell lines and play a role in cell death [9-11]. Specifically, in the lung cancer cell lines A549 and H1299, PKR and pJNK were required for Ad-mda7-mediated cell killing [9,10], whereas in the melanoma cell line MeWo, p38MAPK was required for Ad-mda7-mediated cell killing [17]. Based on these reports, we analyzed for the effect of Ad-mda7-treatment on the expression levels of PKR, p38MAPk and pJNK using Western blotting. Analysis of cell lysates collected at 72 h after treatment showed increased PKR, p38MAPK, and pJNK expression in Ad-mda7-treated ovarian cancer cells but not in PBS-treated or Ad-luc-treated cancer cells (Figure 3). However, we did not observe increased activation of these molecular markers in Ad-mda7-treated normal cells when compared with that in PBS-treated or Ad-luc-treated normal cells.

**Figure 4:**
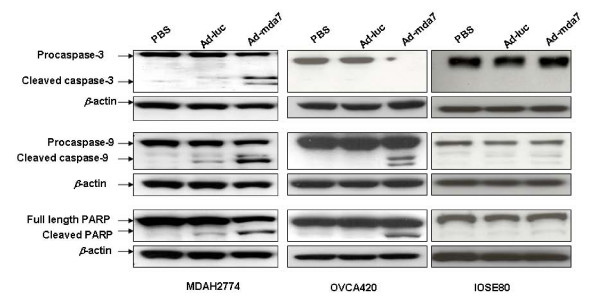
Ad-mda7 activates the caspase cascade in tumor cells. Activation of caspase-9, caspase-3 and PARP indicated by their cleavage products was observed in Ad-mda7-treated tumor cells but not in Ad-mda7-treated normal cells. β-actin was used as a loading control.

Because the previous studies described above showed that PKR, pJNK, and p38MAPK play a role in Ad-mda7-mediated cell-death and because we found that these markers were activated in Ad-mda7-treated ovarian cancer cells, we next analyzed caspase-3, caspase-9, and PARP, all of which are downstream of PKR, p38MAPK, and pJNK, to determine whether they are activated in apoptotic cells. We observed induction of apoptosis as evidenced by activation of caspase-3 and caspase-9 and cleavage of PARP in Ad-mda7-treated MDAH2774 and OVCA420 cells but not in Ad-mda7-treated IOSE-80 cells (Figure 4). We also observed a slight increase in caspase-3 and caspase-9 activation and PARP cleavage in Ad-luc-treated MDAH2774 cells compared with that in PBS-treated MDAH2774 cells. However, we did not observe activation of these molecular markers in PBS-treated or Ad-luc-treated OVCA420 or IOSE-80 cells. These results show that Ad-mda7 inhibits ovarian cancer cell proliferation by inducing cell cycle arrest at G2/M phase and activates several cell signaling molecules associated with apoptosis, resulting in cell death. However, Ad-mda7-mediated inhibitory activity appears to be reduced or absent in normal cells. The ability of Ad-mda7 to selectively inhibit tumor cells and not normal cells agrees with findings of our previous studies [2,8,12].

### Ad-mda7 inhibits ovarian tumor xenografts in vivo

Previous studies from our laboratory showed that intratumoral administration of Ad-mda7 inhibits the growth of lung tumor xenografts [13,14]. Additionally, we showed that systemic nanoparticle-based delivery of mda-7 inhibits experimental lung metastasis [14]. Based on these reports and our present observation of the ability of Ad-mda7 to inhibit ovarian tumor cell proliferation *in vitro*, we tested the growth inhibitory effects of Ad-mda7 *in vivo*

**Figure 5:**
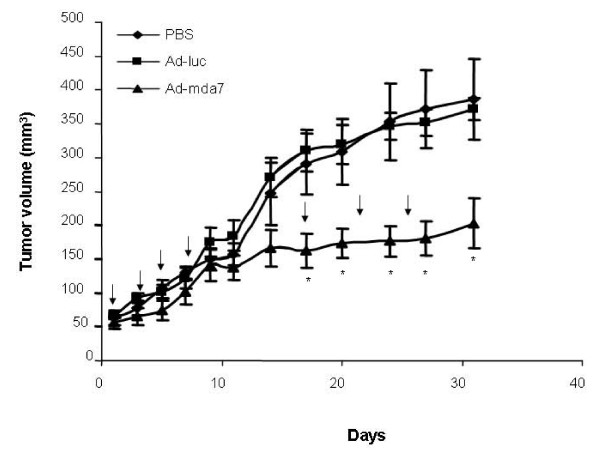
Ad-mda7 inhibits growth of ovarian tumor xenografts in vivo. Subcutaneous ovarian tumors formed after injection of MDAH2774 cells were treated with PBS, Ad-luc, or Ad-mda7 and measured tumor growth using calipers. A significant inhibition of tumor growth was observed in Ad-mda7-treated tumors but not in PBS-treated or Ad-luc-treated tumors. The arrows indicate when treatment was administered. *Significance.

Intratumoral administration of Ad-mda7 in mice bearing subcutaneous tumors formed after injection of MDAH2774 cells subcutaneous MDAH2774 tumors resulted in significant tumor-growth inhibition when compared with intratumoral administration of PBS or Ad-luc (*p *< 0.05; Figure 5). Growth inhibition began on day 18 after initiation of treatment and continued until day 32, at which time we stopped the study. We did not observe significant tumor inhibition in Ad-luc-treated mice compared with that in PBS-treated mice.

**Figure 6:**
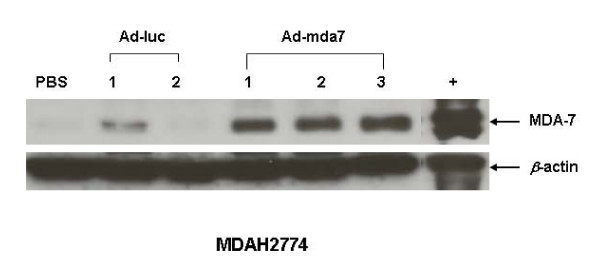
Ad-mda7-mediated tumor growth inhibition is due to MDA-7 protein expression. Tissue lysates prepared from PBS-treated, Ad-luc-treated, and Ad-mda7-treated subcutaneous tumors were subjected to western blotting and detected for MDA-7 protein. Exogenous MDA-7 protein expression was detected in Ad-mda7-treated tumor tissue lysates but not PBS-treated tumor lysates. A non-specific band in one sample of Ad-luc-treated tumor lysate was observed. β-actin was used as a loading control.

Analysis of ovarian tumor tissue lysates showed that the growth inhibition we observed in Ad-mda7-treated mice was caused by MDA-7 protein expression. Specifically, Western blot analysis showed exogenous MDA-7 protein expression in Ad-mda7-treated tumor tissue lysates as evidenced by intense protein banding (Figure 6). We detected some protein banding, albeit at baseline levels, corresponding to MDA-7 protein expression in PBS-treated and Ad-luc-treated tumor lysates. Because *in vitro *studies did not show endogenous MDA-7 protein expression in MDAH2774 cells, we believe that the protein banding seen in the control lysates was probably nonspecific bands that are reacting to the polyclonal antibody against MDA-7. An alternate explanation is that the MDA-7 antibody also detects mouse MDA-7 protein expression, because the subcutaneous tumor xenograft grown in mice comprised of the human ovarian cancer cells intermixed with host cells, including fibroblasts, stromal cells, and endothelial cells. We are currently studying these possibilities in our laboratory.

**Figure 7:**
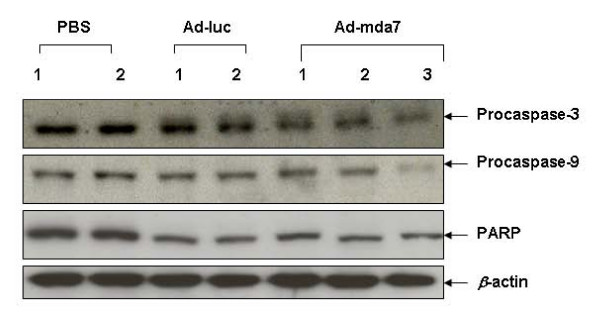
Ad-mda7 activates the caspase cascade in vivo. Activation of procaspase-3, procaspase-9, and PARP expression was observed in both Ad-luc-treated- and Ad-mda7-treated tumor tissue lysates compared to PBS-treated tissue lysate. However, the activation of these molecular markers was higher in Ad-mda7-treated tumor lysates compared to Ad-luc-treated lysates. β-actin was used as a loading control.

We also found that MDA-7 protein expression was associated with detection of decreased procaspase-3, procaspase-9, and PARP expression in Ad-mda7-treated tumor tissue lysates compared to expression levels of these markers in PBS-treated tumor tissue lysates (Figure 7). Surprisingly, we observed lower expression of these markers in Ad-luc-treated tumor tissue lysates than in PBS-treated lysates. However, the decrease, at least for procaspase-3 and procaspase-9, was greater in Ad-mda7-treated lysates than in Ad-luc-treated lysates. Thus, our *in vivo *results showed that Ad-mda7 can effectively inhibit ovarian tumor growth and induce apoptosis similar to that observed in our *in vitro *studies.

## Discussion

In the present study we investigated the antitumor activity of mda-7/IL-24 against human ovarian cancer cells and compared to normal ovarian epithelial cells *in vitro *and the mda-7-mediated growth inhibitory effects on human ovarian tumor xenograft mouse models *in vivo*. For this purpose we used an adenoviral vector carrying the mda-7 gene (Ad-mda7). *In vitro *studies showed Ad-mda7 selectively inhibited the growth of ovarian tumor cells but not normal ovarian epithelial cells. Associated with Ad-mda7-mediated growth inhibition was the marked increase in the number of tumor cells in the G2/M phase of cell cycle indicating cell cycle arrest. The ability of Ad-mda7 to selectively inhibit ovarian tumor cell growth in the G2/M phase with no effect on normal cells concurs with our previous reports on the inhibitory effects of mda-7 on other human tumor cell types [2,4,8,12,15].

Studies in lung cancer and melanoma have previously shown Ad-mda7-mediated tumor cell death occurs by activation of PKR, p38MAPK and pJNK leading to activation of the caspase cascade [7,9-11]. Analysis for activation of these molecular markers in the present study showed increased PKR, p38MAPK and pJNK expression in Ad-mda7-treated ovarian tumor cells but not in normal cells. Furthermore, activation of caspase-9, -3 and PARP was also observed in Ad-mda7-treated ovarian tumor cells but not in normal cells. Our results show that Ad-mda7 induces cell death of ovarian cancer cells by activation of the cell death signaling pathways, an observation that has not previously been reported for ovarian cancer.

Since *in vitro *results do not always correlated with *in vivo *studies, we investigated the growth inhibitory effects of Ad-mda7 using ovarian tumor xenografts established in nude mice. We observed Ad-mda7 significantly inhibited the growth of subcutaneous MDAH2774 tumors. MDA-7-mediated growth inhibition *in vivo *correlated with activation of molecular markers such as caspases that was also observed *in vitro *in Ad-mda7-treated tumor cells. The ability of Ad-mda7 to inhibit the growth of ovarian tumor xenografts is not surprising and concurs with our previous findings on lung tumor xenografts [13]. However, the Ad-mda7-mediated inhibitory effect on ovarian tumors observed in the present study was more significant than that observed previously in lung tumor xenografts [13].

Although we showed in this study that mda-7 gene delivery using an adenoviral vector can inhibit subcutaneous ovarian tumor growth and established a proof of concept, the reality is that ovarian tumors grow inside the abdomen, so the therapeutic effect of Ad-mda7 must be tested in an intraperitoneal tumor model. However, reports have shown that ovarian tumor cells growing in the peritoneal cavity are not efficiently infected with adenoviral vectors carrying therapeutic genes for several reasons, including low surface expression of the adenoviral receptors by tumor cells, interference between the adenovirus and tumor cells by ascitic fluid, rapid clearance of the adenovirus, and induction of host immunity against the viral proteins [16,17]. To overcome some of these limitations, investigators have used targeting strategies that have shown limited success [18,19]. A recent study by Mahasreshti et al. [5] showed that targeted Ad-mda7 delivery can inhibit ovarian cancer growth and prolong the duration of animal survival when compared with that using a nontargeted adenoviral vector carrying the mda-7 gene. However, in that study, the animals received treatment only twice, raising the possibility that when administered repeatedly, a targeted adenoviral vector can become ineffective because of clearance of it by the host immune system. Additionally, in the same study, the researchers administered mda-7 to tumor-bearing mice at earlier time points (days 2 and 14 after tumor cell inoculation) at which time there may not have been enough ascitic fluid to interfere with adenoviral infection.

Based on these reports demonstrating the potential problems of using adenovirus-based therapy for ovarian cancer, an alternate is the use of lipid-based nanoparticles for the treatment of intraperitoneal ovarian tumor-bearing mice. The rationale for testing nanoparticle-based mda-7 therapy are that they are less immunogenic and stable for extended periods of time (6-24 h) *in vivo *compared to adenovirus-based therapy. Furthermore, studies from our laboratory have shown that the lipid-based nanoparticles are effective gene delivery vehicles when administered systemically [14,15]. We in the laboratory are currently testing the antitumor properties of mda-7 contained in lipid-based nanoparticles for the treatment of intraperitoneal ovarian tumor-bearing mice. It is anticipated that these studies will provide a basis for future preclinical studies.

## Conclusion

Our study demonstrates Ad-mda7 can selectively and effectively inhibit ovarian cancer both *in vitro *and *in vivo *and is therapeutic agent for ovarian cancer. However, delivery of mda-7 using alternate gene delivery vector systems such as nanoparticles is required to achieve effective control of ovarian tumor growth in the abdominal cavity.

## Methods

### Cell lines and cell culture

The human ovarian cancer cell lines MDAH2774 and OVCA420 were grown as described previously [20]. IOSE-80 human normal ovarian epithelial cells were provided by Dr Gordon Mills (The University of Texas M. D. Anderson Cancer Center, Houston, TX).

### Adenovirus vector

The replication-defective Ad-mda7 vector was constructed and purified as previously described [8,13]. Briefly, replication-deficient human type 5 adenoviral vectors were constructed to express either mda-7 (Ad-mda7) or luciferase (Ad-luc) genes linked to an internal cytomegalovirus immediated-early promoter and followed by an SV40 polyadenylation signal. The viruses were propagated in HEK293 human embryonic kidney cells and purified by column chromatography.

### Cell viability and cell cycle assay

Tumor and normal cells (10^5^) treated with phosphate-buffered saline (PBS), an adenoviral vector carrying the luciferase gene (Ad-luc), or Ad-mda7 (3000 vp/cell) were subjected to cell viability and cell cycle analysis as described previously [8,12,13]. Cell viability analysis was performed on day 3 and day 5 after treatment, whereas cell cycle analysis was performed on day 3 after treatment. Treatment was performed in triplicate, and experiments were repeated at least twice to ensure reproducibility and statistical significance. The results presented are representative of one experiment.

### Western blot analysis

Tumor and normal cells (10^5^) treated with PBS, Ad-luc, or Ad-mda7 were harvested at 24, 48, and 72 h after treatment and subjected to Western blotting [8,12,13]. The following primary antibodies were used in the Western blot analysis: total double-stranded RNA-dependent protein kinase (PKR), p38MAPK, phospho-specific c-Jun N-terminal kinase (pJNK), and caspase-9 (Cell Signaling, Boston, MA); caspase-3, poly(ADP-ribose) polymerase (PARP), and β-actin (Sigma Chemical Co., St. Louis, MO); and MDA-7 (Introgen Therapeutics, Inc., Houston, TX).

### In vivo studies

Female athymic BALB/c female nude mice of 4–6 weeks age were purchased from Charles River Laboratories (Willimington, MA). Mice were housed in sterile pathogen free environment and fed *ad libitum*.

Prior to start of the experiment mice were subjected to whole body cesium radiation (350 rads) to enhance uptake of human xenograft tumor cells. Twenty-four after radiation the mice were injected with MDAH2774 tumor cells (5 × 10^6^) subcutaneously into the lower right flank (*n *= 18). The mice were separated into groups by treatment (six each for PBS, Ad-luc, and Ad-mda7) and administered treatment when their tumors reached 40 to 50 mm^3 ^in volume. Treatments were administered intratumorally for all groups on days 1, 3, 5, 7, 17, 20, 24, 26, and 32. Ad-luc and Ad-mda7 doses administered were 5 × 10^9 ^vp/dose. Tumor growth was measured two to three times per week as described previously [13,15]. The animals were killed by CO_2 _inhalation at the end of the experiment per institutional approved guidelines. The experiments were carried out twice; data from one representative study are presented. All of the in vivo studies conducted were approved by the institutional animal care and welfare committee and performed according to N.I.H. guidelines.

For molecular analysis of MDA-7, procaspase-3, procaspase-9, and PARP expression in Ad-mda7-treated tumor tissues, subcutaneous ovarian tumor tissue samples were harvested from mice upon termination of the study and snap-frozen in dry ice. Samples were subsequently ground using a homogenizer, and tissue lysates were prepared and analyzed for expression of the indicated molecular markers using Western blot analysis as described previously [21].

### Statistical analysis

All of the experiments were performed twice, and experimental results were analyzed for statistical significance using Student's *t*-test and analysis of variance. Values of *p *< 0.05 were considered statistically significant.

## Competing interests

The author(s) declare the present study was conducted in part by receiving funding from Introgen Therapeutics, Inc., Houston, TX, USA. Dr. Ramesh is a consultant for Introgen Therapeutics, Inc., Dr. Chada is an employee of Introgen Therapeutics, Inc.

## Authors' contributions

B.G. and M.S. were responsible for conducting the in vitro and in vivo studies. S.C. participated in the design of the study, interpretation of the results and provided the adenoviral vectors. R.R. conceived of the study, and participated in its design, and coordination. All authors read and approved the final manuscript.
